# Refugee and migrant women's engagement with sexual and reproductive health care in Australia: A socio-ecological analysis of health care professional perspectives

**DOI:** 10.1371/journal.pone.0181421

**Published:** 2017-07-20

**Authors:** Zelalem B. Mengesha, Janette Perz, Tinashe Dune, Jane Ussher

**Affiliations:** 1 Translational Health Research Institute (THRI), School of Medicine, Western Sydney University, Penrith, New South Wales, Australia; 2 School of Science and Health, Western Sydney University, Penrith, New South Wales, Australia; Harvard Medical School, UNITED STATES

## Abstract

**Background:**

In Australia only 2.2% of published health research has focused on multi-cultural health despite the increase of culturally and linguistically diverse populations. Research on the perceptions and experiences of health care professionals (HCPs) in engaging with refugee and migrant women is also lacking. Given the integral role of HCPs in providing sexual and reproductive health (SRH) care for these populations, an understanding of the challenges they experience is required. Therefore, this study sought to examine the perspectives and practices of Australian HCPs with regard to the provision of SRH care for refugee and migrant women.

**Methods:**

Employing qualitative methods, twenty-one semi-structured interviews were conducted with HCPs representing various professions, work experiences, cultural backgrounds, age and healthcare sectors. The interviews were analysed using thematic analysis and the socio-ecological model was utilised to interpret the data.

**Results:**

The complexities of HCP’s engagement with refugee and migrant women were identified in three major themes: *Being a Migrant*; *Gender Roles and SRH Decision-making*; and *Women in the Healthcare System*. HCPs discussed the impact of accessing SRH care in women’s country of origin and the influence of re-settlement contexts on their SRH knowledge, engagement with care and care provision. Perception of gender roles was integral to SRH decision-making with the need to involve male partners having an impact on the provision of women-centred care. Barriers within the healthcare system included the lack of services to address sexual functioning and relationship issues, as well as lack of resources, time constraints, cost of services, and funding.

**Conclusion:**

Australian HCPs interviewed reported that migrant and refugee women do not have appropriate access to SRH care due to multifaceted challenges. These challenges are present across the entire socio-ecological arena, from individual to systemic levels. Multiple and multidimensional interventions are required to increase SRH utilisation and improve outcomes for refugee and migrant women.

## Background

Sexual and reproductive health (SRH) is “a state of complete physical, mental and social well-being in all matters relating to the reproductive system” [1]. Utilisation of SRH care is associated with improved nutrition, mental health, and positive social and economic outcomes for women, in addition to clinical benefits such as reduced rates of sexually transmitted infections (STI) and unplanned pregnancy [2, 3]. Despite SRH being an important aspect of women’s quality of life, the utilisation of these services by refugee and migrant women is low, leading to negative SRH outcomes [4, 5]. For example, Sebo and colleagues [6] found that utilization of family planning methods and STI prevention were low among undocumented migrant women. Refugee women have also been shown to experience higher rates of unplanned pregnancy compared with women of host countries [7] due to lack of appropriate health information and low utilisation of contraceptives [8]. The World Health Organisation (WHO) has reported that migrant women, including asylum-seeking and refugee women, have a higher risk of experiencing unwanted pregnancy, induced abortion and obstetric complications than women in the host population [9]. This may have significant consequences on the physical, psychological and social health and wellbeing of women and their families [10].

In this paper, the term ‘refugee and migrant women’ is used to refer to refugee and migrant women from culturally and linguistically diverse backgrounds. Previous studies of refugee and migrant women’s SRH have concentrated on exploring views and experiences of women regarding maternity care [11]; uptake of antenatal care [12]; childbirth experience [13, 14]; postpartum care [15]; prenatal testing [16]; and reproductive health [17]. Studies have also provided an understanding of how refugee and migrant women negotiate to address their SRH needs. For example, Hach [18] found that pre- and post-migration experience, lack of understanding about available services and difficulty navigating the healthcare system impact refugee and migrant women’s engagement with SRH care. In addition, Pascale and colleagues [17] stated that refugee and migrant women negotiate their SRH through complex social realities including the loss of social networks as well as cultural and religious factors. Other researchers also found that cultural barriers related to discussing sexuality and sexual health impacted women’s access to SRH services [10]. Whilst these challenges from the end-user perspective are recognised, research into the perceptions and experiences of HCPs in engaging with refugee and migrant women seeking SRH care is lacking [19]. A better understanding of HCP’s perceptions of the challenges experienced by refugee and migrant women and the challenges HCPs themselves experience in providing SRH care to refugee and migrant women is needed to implement appropriate interventions and address women’s SRH needs.

Previous studies have shown the complexities of providing health care to refugee and migrant women. For example, women who experience communication barriers are less likely to receive adequate counselling and culturally sensitive care [20], which may result in lower satisfaction with the care [21]. In addition, HCPs may need more time with refugee and migrant women due to the use of interpreters and the time it takes to build rapport [20]. HCP’s lack of cultural competency can affect their interest and satisfaction with providing SRH care to these women [22]. SRH care provision to refugee and migrant women can be much more complex due to the sensitive and culture-bound nature of SRH [23, 24]. For instance, refugee and migrant women may not be comfortable with discussion and disclosure of their SRH with health professionals, making it difficult to understand their needs [22]. Similarly, healthcare professionals may lack competency to initiate the SRH discussion when serving refugee and migrant women [22]. As such an in-depth understanding of the complexities of engaging with refugee and migrant women seeking SRH care from the perspectives of HCPs is needed in fulfilling their needs and aspirations.

Only a handful of researchers have examined the provision of SRH care to refugee and migrant women from HCP’s perspectives with a focus on the provision of family planning, SRH and maternity care [19, 25, 26]. This limited research provides insights into the challenges HCPs themselves experience at four levels of the socio-ecological arena. For example, at the individual level the women’s past experience and misconceptions about family planning may present challenges to HCPs [19, 26]. At the interpersonal level, HCP’s lack of cultural competency, communication difficulties, cultural difference and misunderstandings and misconceptions were identified to influence the provision of family planning [19, 27]. Organisational level barriers included a lack of training and protocols guiding the provision of health care and unfamiliarity of the women with the health system [25]. At the societal level, gender norms and lack of health insurance coverage potentially compromised refugee and migrant women’s access to SRH care [27]. It is not fully understood how these challenges and barriers affect the provision of SRH care to refugee and migrant women, a gap in the literature that the present study will address.

To explore this topic the research was directed by the following research questions: What are the HCP’s perceptions of challenges and barriers that influence refugee and migrant women’s access and utilisation of SRH as well as care provision in Australia? What are the SRH policy and practice implications of these challenges and barriers for refugee and migrant women?

## Methods

### Participants and recruitment

A qualitative research approach involving one-to-one semi-structured interviews was employed to understand the perceptions and experiences of HCP’s in providing SRH care to refugee and migrant women. Twenty-one HCPs who had experience in providing SRH care to refugee and migrant women were recruited nationally through nursing and public health professional associations, newsletters, email lists, family planning clinics, and snowball sampling. To achieve a broad sample and enhance credibility and richness of the data, interview participants were purposely selected across professional groups, cultural backgrounds and experience with SRH care provision (e.g., years as a HCP, number of refugee and migrant women seen daily) (Table 1). Participants ranged from 32 to 70 years old, with a mean age of 50.6. All were female, twelve were born outside of Australia; and five had a primary language other than English. The HCPs interviewed came from the following professional groups: nurses (8), general practitioners (GP) (5), health promotion officers (5), sex therapists (2) and midwives (1). Participants worked across different sectors of the health system including public hospitals, private and Family Planning New South Wales clinics and non-governmental organisations (NGO), with an average work experience of 21 years (range 2–41). Ethical approval was received from the Human Research Ethics Committee of Western Sydney University with approval number H11034 and informed consent was obtained from all individual participants included in the study.

**Table 1:** Socio-demographic characteristics of the participants.

Variable		(n = 21)
**Country of birth**	Australian born	9
	Foreign born	12
**Age (Year bracket)**	30–45	7
	46–55	6
	56–70	8
**Primary language**	English	16
	Other	5
**Profession**	GP	5
	Nurse	8
	Health promotion officer*	5
	Sex therapist Midwife	2 1
**Sector of work**	Public	5
	Private	2
	Public and private	4
	Non-profit/NGO	10
**Years of experience**	1–10	5
	11–20	9
	21 and above	7

* includes bilingual health educators and health educator managers

### Procedure

The first author conducted the interviews between August 2015 and January 2016, using an interview schedule developed from the findings of a systematic review on the views and experiences of culturally and linguistically diverse women in accessing SRH care in Australia [28]. The interview explored 1) perceived SRH needs of refugee and migrant women; 2) perceptions on refugee and migrant women’s SRH knowledge and utilisation of SRH services; 3) barriers to accessing SRH care by refugee and migrant women; 4) challenges to SRH care provision for these women; 5) personal experiences with the use of interpreters; and 6) HCP's perspectives on how to improve the delivery and utilization of SRH services for refugee and migrant women. Two pilot interviews were conducted with a nurse and a GP to evaluate the interview schedule, which resulted in some topics being added, such as the meaning of the term “culturally and linguistically diverse background” and the challenges of initiating SRH discussions with refugee and migrant women. The interview guide was inductively refined on the basis of emerging themes throughout the interview process. Except for one interview with a GP which was conducted face-to-face, all the other interviews were conducted over the phone. The interviews were audio-recorded and lasted an average of 50 minutes. After each interview, summary notes were taken to allow emerging insights to be included in the subsequent interviews. For example, gender roles and their impact on women’s SRH decision making and care provision were discussed in the first four interviews and were therefore explored in the interviews that followed.

### Data analysis

The interviews were professionally transcribed and with subsequent integrity checking undertaken for accuracy. The data were then analysed using thematic analysis according to the approach described by Braun and Clarke [29] and the socio-ecological model was utilised to interpret the findings. A systematic approach to exploring the perceptions and experiences of HCPs delivering SRH care to refugee and migrant women is appropriate due to the complexities of engaging with these groups of women. The socio-ecological model was most relevant to this investigation given its emphasis on the synergetic relationship between individuals and their social environment [30]. The model also describes the multifaceted interrelationships between individual (micro), interpersonal (meso), institutional (exo) and societal (macro) level factors that shape health behaviour and its management [31, 32].

The use of a socio-ecological framework for this study is justified by the following observations. Firstly, refugee and migrant women’s engagement with SRH care can be influenced by multiple factors and therefore interventions to improve access should consider the interrelationship between the four levels of the model [33, 34]. Second, there is a dynamic interrelationship between women and their environment. As such social, physical and political environments can influence their access to SRH care and similarly the SRH needs and experiences of the women can influence their environments [35]. Finally, refugee and migrant women’s interactions with the environment which take place at individual, group or community levels can be both a source of weakness and strength in accessing SRH care [33]. This model therefore aids in identifying the individual, interpersonal, institutional, and societal level factors that may influence refugee and migrant women’s access and utilisation of SRH care as well as SRH care provision in Australia by HCPs.

The socio-ecological model has been used elsewhere to understand barriers to accessing health care for migrant populations. For example, Shtarkshall, Baynesan and Feldman [32], applied this model to explore the issues Ethiopian migrants face in accessing effective health care services in Israel. Others also used it to explore the perspectives and experiences of HCPs delivering health services to migrants in Northern Sweden [36], Here the model was used to interpret the perceptions and experiences of HCPs providing SRH services for women with migrant and refugee backgrounds in Australia.

Data analysis was purely inductive by which codes and themes were identified from the data and not from the socio-ecological framework or other theory. The analysis began with a familiarisation process which involved the first author reading and re-reading the transcripts, in order to develop first order codes such as “Partner influence”, “SRH utilisation” “Resettlement challenges” and “Talking about SRH”. Simultaneously transcripts were read by all authors who collectively contributed to the development of the coding frame. The whole data set was then coded using NVivo, a computer software package which facilitates organisation of qualitative data, and integrity was checked by the last author who examined the coded data and provided feedback. This was followed by summarisation of the coded data set in relation to specific accounts from individual participants to identify preliminary themes. Through a process of attentive discussion and decision making between all the authors, preliminary themes were then grouped into conceptual themes such as "Being a migrant", "Gender roles and SRH" and Women in the Healthcare System" through a process which involved examining patterns, commonalities and differences across the data. This process enabled the authors to define the core concepts and identify unique and specific stories across each theme. The socio-ecological model was utilised during this phase, in order to identify and interpret factors that impacted SRH care of refugee and migrant women at individual, interpersonal, institutional and societal levels. Finally the results were written by answering the research questions based on interpretative analysis and argument.

## Results

Three broad themes which HCPs described as challenges to optimal SRH care were identified from the analysis: 1) Being a Migrant; 2) Gender Roles and SRH Decision-making; and 3) Women’s Experience with the Healthcare System. Within each theme a number of factors were reported to influence refugee and migrant women’s access and utilisation of SRH as well as care provision reflecting the four levels of the socio-ecological model. At the individual level, the influence of being a migrant including women's experience of SRH services in their country of origin, SRH knowledge and settlement priorities were perceived to have an impact on refugee and migrant women’s engagement with SRH care and care provision. In addition, gender roles in SRH decision-making was said to influence women’s SRH access and the provision of care at the interpersonal level. At the institutional level, difficulty navigating the healthcare system; limited scope of services in sexuality and relationship areas; cost of services; and waiting times were identified as preventing SRH access and utilisation. Lastly, societal level factors such as the taboo nature of SRH in some cultures, gender norms, lack of resources/funding, national healthcare policy were reported to affect receipt and delivery of SRH care.

**Fig 1:**
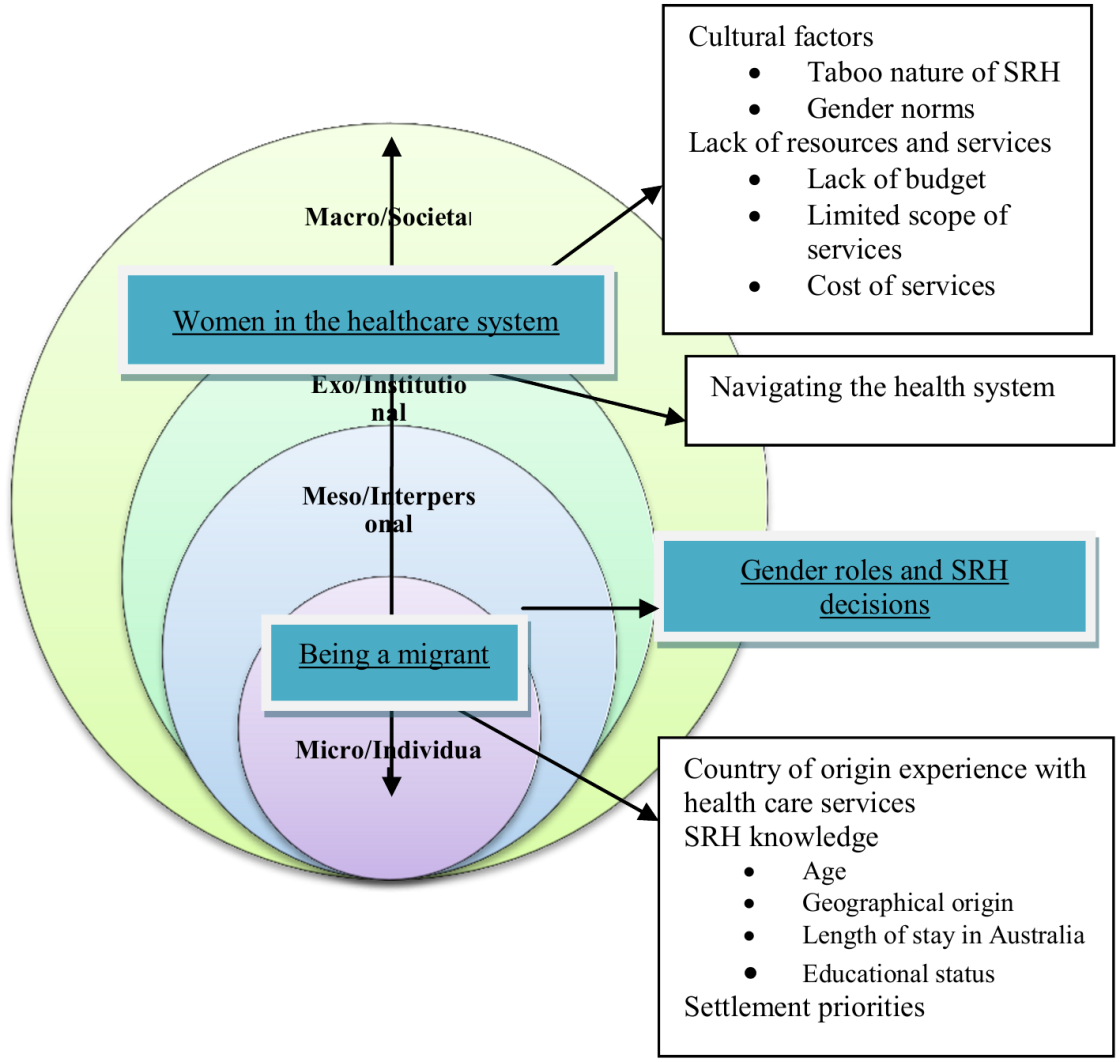
A socio-ecological model of factors influencing refugee and migrant women’s engagement with SRH care.

Fig 1 provides a summary of factors at each level. Even though all the identified factors were aligned with one of the system levels, each factor interacts with the others across levels. In the presentation of the thematic analysis below, we describe how these factors were constructed to impact receipt and delivery of SRH care for refugee and migrant women in Australia. Quotes that highlight the perceptions of HCPs about refugee and migrant women’s SRH are presented with pseudonyms and profession.

### Being a migrant: “We did not have health promotion in our country so we do not see it important here”

In this theme, HCPs discussed individual level factors that describe the experiences of women related to their country of origin experience in accessing and utilising health care, SRH knowledge and settlement priorities which were perceived to influence refugee and migrant women’s engagement with SRH in Australia.

#### Country of origin experience: “They're used to just [accessing] hospital setups”

Many of the HCPs described refugee and migrant women as a heterogeneous group with different migration histories and varying degrees of knowledge, attitudes and experiences in accessing health care. According to the HCPs, women’s experiences in accessing health care in their country of origin influenced their access and utilisation of care after resettlement in Australia. For example, participants reported that women had not been exposed to some SRH services in their country of origin (e.g., cervical screening), or to SRH promotion and, therefore, may not seek them out or know they exist in Australia: “You know in our country we did not have health promotion so we do not see it important here”. (Fatuma, bilingual health educator); “They’re not aware that we have those [screening] services available, especially if those types of services were not available in their own country” (Briggs, Nurse). This suggests that refugee and migrant women may lack a point of references for the service they need to look for in Australia, which may act to constrain their utilisation of SRH care: “They do not know which services to look for…my experiences working with migrant/refugee population is much lower level of access and uptake of services” (Lam, GP).

For some of the health care providers, the availability and structure of women’s health services within migrant women’s home countries were perceived as influencing their health seeking behaviour in Australia: “Even going to see a GP is strange for these [refugee and migrant] people…they're used to just [accessing] hospital setups, or they don't know, or they think they have to pay when they go to hospital” (Hannan, GP). Another practitioner added: “Because they came from different health system[s], where health preventative (sic) is not available, [they] normally go to health services when they get sick” (Sam, Nurse). This implies that the individual level experiences of being a migrant can compromises refugee and migrant women’s participation in preventive services such as cervical and breast cancer screening despite these services being freely available in Australia: “I do find that on the whole refugee and migrant women are less likely to seek screening services than Australian born women” (Tania, GP).

Educating refugee and migrant women was also perceived to be difficult due to the women's limited experience of accessing screening services in their country of origin, as a nurse working in refugee health explained:

*I think there are challenges there just in terms of letting people know that these services are available for them and that it's important for them. Because the countries that they’ve come from, they probably wouldn’t see the general routine screening that we offer in Western countries. They just don’t have the money in their countries to do it. So their mums didn’t do it and their mum's mums didn’t do it, so why should they do it?, which is completely understandable*.

(*Lucy, Nurse*)

As a result, many HCPs suggested the importance of having information about how refugee and migrant women’s previous experience with health systems influences their expectation and access to SRH care.

#### Women's SRH knowledge: "It is quite limited"

HCPs reported that the impact of being a migrant was reflected in women’s knowledge of SRH care. Refugee and migrant women were described as having “quite limited”, “low” or “less knowledge” about SRH and “not [being] aware of many contraceptive options” compared to Australian born women: “My Australian clients have relatively good ‘know-how’ about sexual health, contraception and also the whereabouts of places when they seek help compared to migrants” (Vergenea, Nurse).

Lack of knowledge can have implications for care provision where longer consultation times (standard consultation times in Australian with a medical practitioner are approximately 14 minutes [37]) may be required because of the need to address a number of issues, as one of the respondents explained: “We spend a lot of time explaining sexual and reproductive health…consultations often begin with discussing the very basics of the female reproductive system in my work primarily with Arabic and Iranian women” (Michelle, Midwife). Healthcare professionals emphasised the need to start from scratch and cover several SRH topics when consulting with refugee and migrant women, which takes longer than standard consultation times. Others stressed the need to have “repeated consultations” with these women to increase their SRH knowledge and build their confidence and trust in SRH care.

HCPs discussed several possible reasons for refugee and migrant women’s poor SRH knowledge. For example, Zoi (health education manager) explained that “sexual [and] reproductive health is a topic that is rarely discussed and talked about with in the family circle-there are lots of [knowledge] gaps". In addition, HCPs perceived that resettled women had little opportunity to learn about SRH as “sexual education is not really given such a priority in the developing countries” (Aisha, GP). Even after resettlement in Australia, “information around reproduction and sexual health isn't of high priority as there are more pressing concerns such as—housing, food, safety” (Kokob, health promotion officer). Some participants also said that refugee and migrant women may miss opportunities to learn about SRH due to HCP’s assumptions, as a health promotion officer explained:

“Quite often the topic is not even started by the health professional, assuming that they would have knowledge and they can take care of that part" (Christine, bilingual health educator).

While SRH education and awareness is required for refugee and migrant women in Australia, HCPs suggested that a one-size-fits-all approach is neither ideal nor effective given the heterogeneous nature of refugees and migrants in Australia. For example, two nurses working in family planning clinics explained that refugee and migrant women’s SRH knowledge differed by country of origin and age:

*I've looked after women who have come over from the Arab Emirates, and they have, really, very good knowledge. They have a lot of understanding of how their bodies work. They know about pills, and they're often using the pill already before they come. On the other hand, there's a group of Korean women that come and see us in our Fairfield clinic. They have very little knowledge of contraception*.

(*Chloe, Nurse*)

*It depends on their age…so young Arabic women, from 30 to 40-year-old, seem to have quite a good level of knowledge about that sort of thing. Older women, again, not so much*.

(*Amy, Nurse*)

According to the HCPs, women’s length of stay in Australia and educational status also influenced SRH knowledge: “Women who've been here for a long time generally seem to have better awareness than someone of a refugee background who's recently arrived” (Amy, Nurse); “SRH knowledge varies hugely across same nationality dependent on general education” (Catharine, Nurse). This suggests that providing SRH education to this heterogeneous group can be challenging for HCPs as it may be difficult to ascertain their level of knowledge or how much they need to know: “How can we educate them [refugee and migrant women] in such a way that it's not patronising and the ones who know some stuff, don't get completely bored” (Holly, health promotion officer). Consequently, HCPs emphasised the need to evaluate women’s knowledge during consultation times and provide need-based counselling/education that fits with the women’s level of knowledge and interests:

*I think some women have really high levels of knowledge and some women have not very much at all. So I suppose it's just we have to judge our consultations based on their knowledge. If they need to know the absolute basics, then we do that. Maybe we do a contraceptive consultation over several consults until she feels that she knows what's going on*.

(*Chloe, Nurse*)

These findings highlight the need for HCPs to be flexible based on need with regard to the provision of SRH education and support for refugee and migrant women.

#### Settlement priorities: “Well, they really have other burning issues to address”

Even though refugee and migrant women are offered a range of SRH services in Australia, HCPs reported that that the demands of resettlement are generally prioritised over SRH issues, especially for recently arrived women. Focus is given to fulfilling competing practical and social settlement needs such as housing, employment, income, attending English language classes and childcare responsibilities:

*Well, they really have other burning issues to address. So their burning issues are housing, where to live with their family, how to live, and the schools for kids, learning the language, employment. All these things are more prioritised that sexual reproductive health*.

(*Zoi, health education manager*)

Consequently, these priorities may compromise refugee and migrant women’s overall engagement with SRH care as they may “not look for information or use preventive healthcare services when they feel constrained by demanding settlement needs”.

Further, refugee and migrant women’s resettlement context may make planning health promotion activities difficult for HCPs. HCPs said that women often have challenges finding free time for health issues, and they do not turn up for health education appointments even after accepting invitations and receiving reminders:

*Life is busy so women financially support their families, they work or they have other commitments so it is very difficult to organise a session with them. From my experience we invite them and they say ‘we are coming’…some of them they don't come and some of them they come late. This is the problem we have, for example if you intended to have around 15 women in the group session you'd end up having eight or nine even though we call and remind them*.

(*Fatuma, bilingual health educator*)

Consequently, refugee and migrant women were reported to reactively access SRH care rather than engaging with preventive services like early antenatal care or reproductive screening:

*What we've seen in our experience is that these vulnerable groups access the medical assistance or care only in the case of emergency, when they really have to. But there is not much focus on prevention or really learning about things or accessing the information—how I can improve my life or my health because of the other burdening priorities—housing, English, school for kids, employment etcetera*.

(*Zoi, health education manager*)

Being a migrant can therefore redefine women’s understandings and access to SRH after resettlement. However, some cultural aspects of their country of origin may persist and influence refugee and migrant women’s engagement with SRH.

### Gender roles and SRH decisions: “I have to get the permission of the husband”

The interpersonal level theme identified in the analysis focused on the impact of gender roles on refugee and migrant women’s SRH decision-marking. HCPs discussed their concerns over what they described as a patriarchal gender structure that gave husbands power over their wife’s sexuality and her ability to access contraception care. For instance, in discussing how some men controlled sexual activity within a marriage, one of the nurses explained: “Women are not empowered to make decisions about their sex life and some unable (sic) to deny husband intercourse even if painful” (Lily). Participants also stated that despite some women having knowledge about contraception, they were not able to make decisions when to have a baby and how long to space pregnancies as this was constructed to be the responsibility of the husband:

*I remember doing some sessions with women about contraception and they actually knew a lot of stuff. Then I said ‘so you know all this stuff, but you're not using contraception. Why not?’ They said ‘because it's not our choice to make. It's our husband's choice’. So our husbands say ‘no, I don't want you—I want you to have children. I don't want you to decide when the child is going to be born’*.

(*Holly, health promotion Officer*)

For some HCPs this was particularly evident in comparison to Australian-born women:

*In Australia it can be assumed that a man would use condoms himself or will have less control on women, on his wife's contraceptive choice whereas from the migrant and refugee women, their family is different. The man would have more control on what he wants as a choice of contraceptive than the women*.

(*Christine, bilingual health educator*)

These findings may suggest that refugee and migrant women in Australia experience reproductive coercion resulting in unintended pregnancies. As a health promotion officer explained, husbands limit the women's authority over their fertility to get them pregnant against their will:

*I know of women who have said ‘I'm only going to have two children’, and then I see they're pregnant again. I go ‘what happened?’ She said ‘my husband. I didn't have a choice’. So I'm just reading between the lines in that instance but definitely women have said to me it's not my choice. I can't make that decision. It's not my decision to make. My husband makes that decision*.

(*Holly*)

HCPs described women attended appointments with their husbands, which HCPs described as a challenge as it impacted on their ability to understand the needs of the women. Male partners were described as dominating during consultations, often leaving women unable to disclose their SRH issues in front of their partner. As Tayla (GP) said, “I have had an Iranian couple where the husband did all the talking and it was difficult to talk (to) the woman. It was very difficult to discuss sex/fertility etc. as the woman would not speak in front of her husband”. In a further account Emma (Sex therapist) told us: “It’s frustrating all around for me in that sense, because I’m getting this filtered version of something when the partner is present”.

In some instances, partners served as interpreters for their wives, because “husbands refuse to allow us [HCPs] to engage an interpreter”. This practice was described as creating a frustration among HCPs, as husband interpreters were perceived to not be honest with the interpretation process: “Appear to know, for example, that she wanted an IUD for contraception, however, as husband has been interpreter and speaks for her and hasn't told her of this [the presence of IUD in the clinic]” (Tayla, GP). Furthermore, HCPs told us that healthcare guidelines require consent to be obtained directly from the client during health care provision. But this was not practical when seeing women from refugee and migrant backgrounds in the presence of their partners, as described by one of the GPs:

*If I want to put a woman on the Pill, for example, I have to get the permission of the husband, or it has to go through the husband. Even if I'm concerned about the woman for some reason, it's the husband [who] gives permission*.

(*Lily*)

This demonstrates that providing SRH care that focuses on the women’s individual unique needs and aspirations can be challenging for HCPs: “It is very difficult to provide confidential care or woman-centred care for reproductive services, as some women will not make decisions or even have discussions without the husband's consent” (Rebekah, health promotion officer). However, from HCP’s narratives, accounts of women resisting these practices of their partner were identified. For example, a number of women were described as wanting to know how they could use contraception without their husband’s knowledge, as illustrated in Holly’s accounts: “One frequently asked question was ‘what kind of contraception I can use that my husband won't know about” (health promotion officer). As a result, HCPs stressed the need for private appointments without the husbands to ensure women have the opportunity to “have confidential conversation” and, “do screening for domestic violence”, sexual health and relationship issues.

Despite the perception that couple-counselling has challenges in providing women-centred care, many HCPs recommended partner involvement in consultations with refugee and migrant women for SRH issues suggesting that men should be involved in contraception consultations with women as contraception is “about both of them” and “a partnership”. This provides an opportunity to provide SRH education to men and women within the consultation, as explained by a GP working in a multicultural family planning clinic:

*If I'm putting in an IUD, they [men] need to use a reliable method of contraception. If they [women] don't want to go on the pill I'll say ‘look, it's your duty to use condoms for three weeks or no sex for three weeks. So you have to help us. How can we do that? Do you—come to an agreement?’ So that way, get them involved, not isolating them*.

(*Selome*)

Further, HCPs indicated that involving men would also help women access SRH care as some women may have financial constraints:

*If we don’t involve the men, then often women won't engage in the services or won't seek out the services because often men, they may hold the purse strings as well for instance. So without their support, women are often, even from a financial perspective, can't actually access the services because they might need funds to do that*.

(*Lucy, Nurse*)

Overall the interpersonal level factor of gender roles in SRH decisions was perceived to be a source of both weakness and strength in accessing SRH care for refugee and migrant women.

### Women in the healthcare system

In this theme, HCP's discussed institutional and societal level challenges refugee and migrant women face in accessing SRH care which include difficulty navigating the healthcare system, limited scope of available services, cost of services, and lack of resources.

#### Difficulty of navigating the health system

HCPs generally considered refugee and migrant women to be unfamiliar with the Australian healthcare system and lacking knowledge about available services including the availability of interpreters, and the whereabouts of centres and facilities that provide SRH care. Tania (GP) describes the impact of these challenges: “You can’t use something if you don’t know it exists and if you don’t know much about something, it is more likely to seem scary too”. This implies that refugee and migrant women need to be reassured about SRH services during resettlement, as HCPs added: “They [refugee and migrant women] need for the service to be validated by peers before they feel comfortable to use them” (Briggs, Nurse). The lack of familiarity with the general health system was seen to impact on refugee and migrant women’s ability to navigate the system:

*Oh, the processes around knowing what is a bulk-billing service—how do I make an appointment -how do you know which healthcare services do what? Do you just go to the GP for everything, or would you go to a specialist service, and how would you know the difference? They wouldn't be able to formulate exactly where they need to go*.

(*Kokob, health promotion officer*)

On the other hand, there was a sense among the HCPs that the health system is difficult to navigate for skilled migrants, let alone for refugee and migrant women who might have language and other barriers. A migrant nurse explained her experience of navigating the system: “As a non-Australian I find it difficult myself at times and I have a healthcare background, have not been through trauma and do not have English as a second language” (Liza).

HCPs provided several reasons for the difficulty of navigating the healthcare system. For example, many interviewees perceived that SRH was a taboo topic for refuge and migrant women. This is reflected in the interviews where a number of HCPs described SRH as a "hidden issue", "banned topic" or, "not openly discussed". As a family planning nurse explained, this resulted in difficulty navigating the system:

*They're not things that the community just discuss openly, so I think because of that it's actually kind of hard to find out what services exist because if you're asking about the services, you're asking about the—you're opening the topic of the sexual and reproductive health issues…if you've got a vaginal discharge I think that's quite difficult to talk about that to somebody, so therefore how do you know where to go? I think it's just about what the issues are that we deal with, make it hard to find out where the services are*.

(*Eva*)

HCPs also described local issues where “sexual and reproductive specific health services are not adequately advertised in various languages, at Public Hospitals” and “(hospital departments) are often not well signposted, they're not signposted in multiple languages, so all of that creates confusion” regarding which service to look for or the institution/department to visit. Additionally, a health promotion officer indicated that difficulty navigating the system was due to the system’s bureaucracy to access care:

*Difficulties of migrant/refugee clients in negotiating the Australian health care system to access what they need…often needing to visit multiple places to get care with minimal communication between involved centres*.

(*Zoi, health education manager*)

Even after getting to the destined health facilities, many HCPs reported the presence of long waiting time to receive care particularly for public health insurance claimable appointments and in public hospitals despite some participants reflecting the presence of flexibility based on need. Consequently, refugee and migrant women experience unintended health outcomes such us unplanned pregnancy:

*We had a lady who had eight children. She was quite young. I think she was 34 or 32. Her and her husband had wanted to have—she wanted to be sterilised. She paid to see a specialist who refused to refer her. So then she came to see us, and we referred her to the local hospital for a sterilisation. The waiting list was 18 months long, and in that time she fell pregnant again. She had been actively trying to prevent another pregnancy, and really knew that she didn't want another pregnancy. Yet the system let her down and she was left pregnant with an unplanned, unwanted pregnancy*.

(*Chloe, Nurse*)

Further, HCPs noted that women often experience care breakdown before fulfilling their SRH needs. A migrant nurse provided her insights about the lack of continuity in SRH care in Australia compared with the UK, despite the perception that the two health systems have some commonalities:

*I think it's easier in the UK in a way for people not to get lost in the system. So in the UK, you're linked in with a GP who is basically your GP for life… but the good thing about that is that there's much greater continuity of care. There will be a midwife and a health visitor attached to that GP. So if a woman gets pregnant, she's got the midwife and once the baby is born there will be the health visitor right up until that kid turns five. Whilst those systems are in place in Australia, it's a lot more ad hoc. It's much easier for people to fall through the cracks here*.

(*Lucy, Nurse*).

Respondents stressed the need for “better communication” between health services within the system and “continued follow up beginning with the Refugee Health Service” to prevent women from dropping out while having unmet healthcare needs that may expose them to unintended SRH outcomes.

#### Limited scope of services and resources

HCPs discussed other institutional level barriers that limit refugee and migrant women’s access and utilisation of SRH care-notably the lack of services. Overall several HCPs believed that SRH care in Australia is inclined towards STI screening and contraception with little attention to sexual functioning and relationship areas:

*Well, sexual health services in Australia, are most of the times provide STD [sexually transmitted disease], sexual transmissible infections and perhaps family planning services. But not so much sexual functioning services where a person can ask about specific questions about sexual functioning. Because the sexual health clinics are really more geared towards the screening of sexual transmissible diseases and the counsellors there don’t have much training in sexual functioning and relational areas*.

(*Emma, Sex therapist*)

Despite this lack of service is not be a barrier specific to refugee and migrant women, it may suggest a lack of sexual health services in Australia for people looking outside of STI treatment, contraception and pregnancy care.

Refugee Health Services are the first point of contact for arrivals on humanitarian program (e.g., refugees, asylum seekers, and women at risk) during the early periods of resettlement. HCPs described these services as focusing on vaccination and screening of common health conditions, with little attention paid to other services women need:

*A lot of those services [Refugee Health] are really just looking at the major blood borne viruses. They're just looking at vaccinations and screening for HIV and TB. They're not actually looking at their day-to-day health—other health services that people might want to access*.

(*Lucy, Nurse*)

In another account, Eva (Nurse) mentioned the lack of SRH services within public hospitals where several refugee and migrant women often visit to access maternity care:

*We don't provide sexual and reproductive health services to any great degree within the public health system in Australia, within the hospital system, which is often where a lot of refugee and migrant women actually meet the health service, maybe when they go to have a baby and go to the hospital clinic for some reason. But they don't necessarily have good SRH services within the hospital system, so it's hard to find a specialist type service I guess*.

(*Eva*)

This account again may reaffirm a lack of sexual health services, mainly within the public health system in Australia. Some HCPs also added that “once women leave refugee health clinic, which is generally after about six months, a significant proportion of them do not go on to access general practice services. So often their sexual and reproductive health needs are not met on an ongoing basis” (Naomi, GP). Consequently, participants recommended the need for SRH integration within the hospital and Refugee Health Services to “address SRH needs early on, when new arrivals come” and assist “them [refugee women] to access family planning services on site”.

The scope of available services for refugee and migrant women was seen as further constrained by the lack of resources. Despite HCPs believing that refugee and migrant women need education that takes into account their socio-demographic and cultural backgrounds to help them make informed choices about their SRH, the majority of interviewees told us they faced resource constraints to provide the SRH education: “The biggest challenge is we have very limited budget and we have to cover all areas of women's health throughout the whole of Victoria” (Zoi, health education manager). Zoi again added that “Due to the budget, we can't employ educators to represent women from all communities that live in Victoria given that there are over 200 different languages”. Similarly, Kokob (health promotion officer) said that “we have very limited resources that are in a number of different languages to access all women that need education.” Due to the sensitive nature of SRH, health promotion officers wanted to provide individualised education to the women. However, Christen (HE) explained that “there's not much funding for us to do one on one session”. This suggests that access to SRH education for refugee and migrant women is constrained: “Many women have had no access to education about sexual and reproductive health and hence are unable to make informed decisions about what is appropriate for them” (Briggs, Nurse).

HCPs also reported that cost of services was a limitation for refugee and migrant women to access a range of services they need. Chloe (Nurse) asserted that “access to IVF and fertility treatments is very difficult for refugee and migrant women due to the cost”. Selome (GP) also added that “Where do they find money to buy IUD?” This was because “some groups of women did not have public health [Medicare] benefits”; “the waiting lists for Medicare based appointments are unreasonable at times; and there was also “a lack of practices that billed directly to the public health system in some areas”. HCPs who were working in private practices also disclosed that they did not commonly see women from refugee and migrant backgrounds:

*Very rare from refugee background, because of where my clinic is—it's in a well to do part of Sydney and I’m also in private practice, so that precludes a lot of—it excludes a lot of a people from a refugee background*.

(*Emma, Sex Therapist*)

Regarding contraception, some organisations tried to help refugee and migrant women by waiving fees and putting free samples for them to use in their practices: “We are very lucky in our organisation that we actually can waive fees for those [women who can’t afford] clients”. (Lucy, Nurse); “We try and have some samples [contraceptives] and help them with it” (Selome, GP). However, this was complicated for HCPs: “We can’t absorb that (fee waiving) anymore” (Selome, GP).

Overall the findings suggest that “Migrant and refugee women do not have equitable access to SRH care in comparison to Australian born women” (Liza, Nurse) due to the multifaceted barriers they experience which involve elements within all levels of the socio-ecological framework.

## Discussion

The 1994 International Conference on Population and Development in Cairo recognised that refugees and migrants have the right to seek and receive SRH information and care before and after resettlement in new countries [38]. Utilisation of SRH is also associated with improved health outcomes for women and their children [2, 3]. Despite Australia’s healthcare system being one of the best performing healthcare systems in the world [39], refugee and migrant women are less likely to have access to SRH information and care than Australian born women [5, 18, 40, 41]. The present study was conducted to explore the complexities and barriers of providing SRH care to refugee and migrant women from the perspectives of HCPs through a socio-ecological lens.

In agreement with previous studies [42, 43], HCPs in this study indicated that refugee and migrant women are a diverse group in relation to their migration history and experience, age, education, culture, socio-economic background, country of origin experience in accessing health care, length of stay in Australia and SRH knowledge. However, common concerns around accessing and utilising the available SRH information and services and SRH literacy were indicated in this study. Whilst knowledge is one of the key components of people’s ability to adopting protective health behaviour [44], HCPs accounts indicate that women from refugee and migrant backgrounds have very limited SRH knowledge including low levels of awareness and familiarity with modern contraceptive options. These results are in line with those of previous studies conducted by Ngum Chi Watts and colleagues [8] and the Multicultural Centre for Women’s Health [5], which showed SRH knowledge deficiency among this group. This knowledge deficit may put refugee and migrant women at greater risk of having an unwanted pregnancy and STIs [45], and highlights that SRH education should be part of early resettlement services for refugee and migrant women [23].

Some previous research in Australia considered migrants as a homogeneous group which makes understanding SRH differences within and among cultures difficult [46]. However, HCP’s perceive that refugee and migrant women’s SRH knowledge and engagement with care varied with their individual level factors such as age, country of origin, length of stay in Australia and educational status. These results support the findings of Rogers and Earnest [47] who reported intergenerational differences regarding reproductive health and contraception knowledge among Sudanese and Eritrean women in Brisbane, and Dawson and Gifford [48] and Hannah and Lê [49], who suggested that time of migration and level of education in a new country correlates with greater sexual health knowledge. This implies that cultural and socio-demographic backgrounds should be considered when providing SRH care and education [8, 50].

Several reports have shown that refugee and migrant women’s previous experiences in accessing health care influenced how they access the services in the new countries [49]. For example, O'Donnell and colleagues [51] reported that migrants, refugees and asylum seekers’ diverse experiences in accessing health care in their country of origins, mainly characterised by a lack of GPs as a first point of contact and access to hospital based specialists without the need to make an appointment, shaped the way they were accessing health care in the UK. These reports affirm the results of the present study where refugee and migrant women’s health seeking behaviour, especially participation in cancer screening and health promotion services, were compromised by their previous experiences. According to the HCPs, women came from countries where Pap smears and other preventive health services provided in Australia were not available. Therefore, they do not know these services exist or reach out to access them. This suggests that women's country of origin experience impacts their SRH choices in Australia which may have negative consequences on their health and agency [10]. This could also be one of the possible explanations for the lower participation rate of women from refugee and migrant backgrounds in cervical screening programs compared to Australian born women [40]. HCPs could benefit from additional information concerning the health systems which refugee and migrant women used to access and the way their country of origin experiences shape their expectations of SRH care and health seeking behaviour in Australia [51]. In addition, migrant and refugee women would need education about how the health system functions and preventive services available to them in Australia [18].

Another important observation by HCPs was that refugee and migrant women’s primary focus after arrival in Australia is on fulfilling resettlement needs such as housing, learning English language, schooling for kids, and achieving family responsibilities. Women also engage in paid work to fulfil the socio-economic demands of resettlement. The triple burden of engaging in resettlement activities, fulfilling family responsibilities and engaging in paid work makes it difficult for refugee and migrant women to consider reaching out for appropriate SRH care and this may compromise their overall health [23, 52]. These results corroborate the findings of O'Mahony and Donnelly [53] and McMichael and Gifford [23], who reported that life contexts of people from refugee and migrant backgrounds impact their ability to access and utilise the available SRH information and care. To close the gap in SRH literacy and utilisation between refugee and migrant women and women of host nations, policies and programs should be concentrating on the women’s social contexts-mainly on their resettlement experiences, family and economical contexts, which shape the daily experiences of resettled refugee and migrant women [23].

Guruge and Khanlou [33] suggest that the health and wellbeing of refugee and migrant women is greatly influenced by their relationship with the family, which can be either a source of strength or a barrier in accessing care for the women. In this study, gender roles were perceived to influence recept and provision of SRH care, with husbands being involved in family planning consultations and decision makings with refugee and migrant women. These results are consistent with those of Newbold and Willinsky [19] who reported that migrant women have little say in contraception decisions. This can have several implications for the women and HCPs. For example, HCPs in this study revealed that women who experience partner control are more likely to hide their contraception from their husbands and this may become a source of conflict and domestic violence when revealed. Furthermore, partner involvement during consultations can make it challenging for HCPs to provide important resources to the women [53] and teach them how to negotiate the use of contraception with their partners [19]. It may also create frustrations among HCPs when partners become interpreters as they may not trust the partner’s interpretation [53]. This suggests that gender roles in SRH decision making can compromise refugee and migrant women’s access to care and the provision of women-centred care despite the fact that some participants recommended partner involvement in SRH care provision to the women [53].

Another important finding was that refugee and migrant women have limited opportunities for accessing SRH care due to the organisational and societal level factors related to the healthcare system. Some of the barriers they face such as cost of services and long waiting time to get care are similar to those experienced by all women, particularly those from refugee and migrant backgrounds [47, 54]. One unanticipated finding was that there is lack of services related to sexual problems and relationship areas which participants described as one of the factors which can contribute to limiting access to SRH care. However, challenges such as navigating the healthcare system and lack of resources in the form of funding to support health education programs for refugee and migrant women and translated materials in their languages bring additional challenges to the process of accessing and receiving SRH care [55, 56].

This study has a number of strengths and limitations that should be noted. The research included HCPs representing various professions, work experiences, cultural backgrounds, age categories and healthcare sectors, and this participant diversity helped to enrich the data as it provided broader insights of the topic from diverse groups. However, people who work closely with the refugee and migrant population such as settlement organisations, refugee mentors, professionals working in refugee health clinics and interpreters were not included. These groups of people may have better understandings why refugee and migrant women, especially recent arrivals, experience challenges when accessing SRH care in their new country. In addition, previous studies reported that refugee and migrant women prefer to see female HCPs for their SRH issues [57], and access to SRH information and service is limited in rural areas of Australia [58]. Nevertheless, in this study, all the interviewees were females working in metropolitan areas despite efforts to include male HCPs and those who work in rural areas. Therefore results should be interpreted cautiously, and future research in refugee and migrant women’s SRH may benefit from including these unreached professional groups.

There are two areas of additional research in refugee and migrant women’s SRH that are needed. First, research from the perspectives of refugee and migrant women concentrating on their experiences of accessing specific SRH services such as family planning, cancer screening and sexual problems is needed to substantiate the findings of this study. Second, general practitioners are the first contact within the Australian health care system when people look for health services. However, in this study, the majority of the participants indicated that GPs have constraints to provide SRH care to refugee and migrant women such as lack of SRH training and competency, not willing to deal with consultations that take longer time and require the use of interpreters. Participants also added that GPs commonly refer the SRH issues of refugee and migrant women to other levels. Consequently, further research is needed regarding the perceptions and experiences of GPs in engaging with refugee and migrant women for SRH issues to assess these claims; better understand their perspectives and provide the necessary support.

## Conclusion

Adopting Bronfenbrenner`s socio-ecological framework in the analysis of HCP's accounts, we were able to identify several barriers that impact refugee and migrant women’s SRH care within the entire socio-ecological environment- individual to systemic levels. Interventions informed by the socio-ecological model need to attend to all levels to improve the women’s access and utilisation of SRH care. The intersections of individual level factors lead to differences in SRH literacy and engagement both within and between cultures of women. As such refugee and migrant women should be approached as individuals, not as a homogenous group during SRH care provision [35]. During consultations HCPs should also put efforts to understand the women’s previous experiences in accessing SRH care and daily life realities during and after resettlement in order to identify barriers and provide appropriate support [23, 35]. Whilst SRH problems occur at the individual level, women receive and engage in care and treatment in a context of interaction with families, in this case with their partners, which constitute the interpersonal level [33]. This suggest the need of couple based family planning education as part of SRH education to improve husband’s approval of family planning and their willingness to meaningfully engage with their partners in relation to contraception matters [59]. Women also need alone time with HCPs to freely discuss their SRH needs and aspirations. Finally at system level, if access to SRH education and service are to be guaranteed for refugee and migrant women, policy makes need to give due attention to retaining and increase funding to refugee health programs, expanding the scope of services, strengthening outreach education services, and ensuring that all women of ages and cultural backgrounds have access to SRH education and care [18, 47, 60].
